# Changing the conversation: impact of guidelines designed to optimize interprofessional facilitation of simulation-based team training

**DOI:** 10.1186/s41077-024-00313-3

**Published:** 2024-10-12

**Authors:** Mindy Ju, Naike Bochatay, Alexander Werne, Jenna Essakow, Lisa Tsang, Mary Nottingham, Deborah Franzon, Audrey Lyndon, Sandrijn van Schaik

**Affiliations:** 1 Division of Pediatric Critical Care Medicine, Department of Pediatrics, University of California, San Francisco, 550 16th St., Floor 5, San Francisco, CA 94143 USA; 2 Karger Publishers, Basel, Switzerland; 3 Department of Pediatrics, Palo, Alto Medical Foundation, Sutter Health, Palo Alto, CA USA; 4 Clinical Pediatrics, Department of Pediatrics, Vanderbilt University, Nashville, TN USA; 5 Pediatric Clinical Nurse Specialist, UC Davis Children’s Hospital, Sacramento, CA USA; 6 Pediatric Clinical Nurse Specialist, Benioff Children’s Hospital San Francisco, San Francisco, CA USA; 7 Rory Meyers College of Nursing, New York University, New York, NY USA

**Keywords:** Interprofessional, Teamwork, Collaboration, Communication, Simulation, Co-facilitation, Co-debriefing

## Abstract

**Background:**

Interprofessional simulation-based team training (ISBTT) is commonly used to optimize interprofessional teamwork in healthcare. The literature documents the benefits of ISBTT, yet effective interprofessional collaboration continues to be challenged by complex hierarchies and power dynamics. Explicitly addressing these issues during ISBTT may help participants acquire skills to navigate such challenges, but guidelines on how to do this are limited.

**Methods:**

We applied an educational design research approach to develop and pilot structured facilitator guidelines that explicitly address power and hierarchy with interprofessional teams. We conducted this work in a previously established ISBTT program at our institution, between September 2020 and December 2021. We first reviewed the literature to identify relevant educational theories and developed design principles. We subsequently designed, revised, and tested guidelines. We used qualitative thematic and content analysis of facilitator interviews and video-recording of IBSTT sessions to evaluate the effects of the guidelines on the pre- and debriefs.

**Results:**

Qualitative content analysis showed that structured guidelines shifted debriefing participation and content. Debriefings changed from physician-led discussions with a strong focus on medical content to conversations with more equal participation by nurses and physicians and more emphasis on teamwork and communication. The thematic analysis further showed how the conversation during debriefing changed and how interprofessional learning improved after the implementation of the guidelines. While power and hierarchy were more frequently discussed, for many facilitators these topics remained challenging to address.

**Conclusion:**

We successfully created and implemented guidelines for ISBTT facilitators to explicitly address hierarchy and power. Future work will explore how this approach to ISBTT impacts interprofessional collaboration in clinical practice.

**Supplementary Information:**

The online version contains supplementary material available at 10.1186/s41077-024-00313-3.

## Introduction

Interprofessional simulation-based team training (ISBTT) has become a common educational strategy to optimize interprofessional teamwork in healthcare [1–5]. Reports in the literature suggest that ISBTT can positively impact attitudes and perceptions of teamwork among healthcare professionals, improve interpersonal, communication, and behavioral skills in both simulated and real-life scenarios and improve patient safety [6, 7]. Yet, despite these positive effects of ISBTT on teamwork and the widespread use of ISBTT, there is also evidence that ineffective interprofessional collaboration and communication continue to compromise safety and quality of patient care [8–13]. Complex hierarchies and power dynamics between different professionals are major factors in impeding effective teamwork in both clinical [14, 15] and ISBTT [16] environments, leading to a growing literature on the importance of speaking up as part of teamwork and teamwork training [17–19]. This has led to the development of new approaches to debriefing and other interventions [20–24] that aim to promote speaking up, yet a few focus on interprofessional teams [25]. Studies examining the role of hierarchy and power in ISBTT are limited to date, but from the few studies published, it seems these factors influence behaviors of facilitators and participants alike [26, 27]. In our recently published scoping review of ISBTT programs, we noted that only 7 out of 58 included articles described pre- or debriefings in which psychological safety was explicitly addressed and even fewer mentioned discussions of power and hierarchy [28]. Our prior multi-institutional research further provides evidence that power and hierarchy are rarely discussed during ISBTT across varying specialties and hospital systems, [29] pointing towards a potential opportunity to augment the impact of ISBTT by incorporating such discussions.

In a previously published case study of seven ISBTT programs across five different hospitals, we noted that interprofessional interactions were rarely the primary focus of debriefing [29]. If interprofessional collaboration was brought up, the discussion was typically brief and superficial, and conversations about medical knowledge, clinical reasoning, and system issues were prioritized. Feedback discussed during the debriefing focused mostly on individuals’ clinical knowledge and/or performance of tasks. In a separate study examining debriefing in our Children’s Hospital ISBTT program, we observed that nurses discussed issues related to team functioning and physician leadership during intra-professional (nurse-only) debriefing, but not during interprofessional debriefings. In subsequent interviews with these nurses, they cited hierarchy, power differentials, and fear of conflict as barriers to sharing concerns across professions [30]. Hierarchy and power differentials are not unique to healthcare, and their negative impact on team performance and team outcomes has been well documented in the organizational psychology and business literature [31]. Reducing power distance, shifting leadership models to shared or collaborative models, and creating a culture of psychological safety are some of the ways in which such barriers can be mitigated [32–35]. Of these approaches, only psychological safety is a well-established concept included in guidelines for ISBTT [36, 37].

Thus, for ISBTT to reach its full potential in improving interprofessional collaboration, training may need to include strategies to reduce power distance, model collaborative leadership, and discuss the origin and impact of power and hierarchy on healthcare teams in a psychologically safe manner [38]. The latter in particular can be challenging for participants and requires thoughtful facilitation [39, 40]. A natural place for such discussions to take place is during the debriefing, after a simulation scenario, where most learning from ISBTT typically occurs [41–45]. During the pre-briefing, when facilitators discuss ground rules and expectations for participants, they can forecast that these topics will be discussed. Best practices for how to facilitate pre- and debriefings have been published and various models exist, [45–49] but most published guidelines do not explicitly discuss how to debrief interprofessional teams, despite common recognition that this is more challenging than for uni-professional teams [27, 36, 50, 51]. The literature on how to facilitate ISBTT thus remains limited, and experts in interprofessional debriefing have called for increased research in evaluating debriefing in ISBTT [27, 52].

To build on the existing literature and provide simulation educators with practical guidelines for adaptation to their own setting, we developed an innovative approach to debriefing after interprofessional team training that empowers facilitators to explicitly discuss team dynamics and processes for the promotion of collaborative skill development [53]. We aimed for the guidelines to promote a truly interprofessional, collaborative approach to ISBTT and help facilitators lead discussions about interprofessional team dynamics (including power and hierarchy) and their impact on teamwork in healthcare. We used educational design research (EDR) methodology to develop and test the guidelines through an iterative process in an existing ISBTT program. In this article, we present our work during the three phases of the EDR, examining through qualitative analysis how facilitators used the guidelines and what factors promoted and impeded their implementation. We also include the final guidelines and recommendations for implementation at other institutions.

## Methodological approach

EDR is increasingly utilized to improve educational practices, [38–40] relying on iterative small-scale testing of potential solutions to a problem. The approach resembles “Plan, Do, Study, Act” (PDSA) cycles, as initially developed for industry and extensively applied to healthcare quality improvement projects [41]. This approach allows for implementation in real-world settings, which is particularly attractive for complex problems in which many contextual factors play a role [40].

**Fig. 1:**
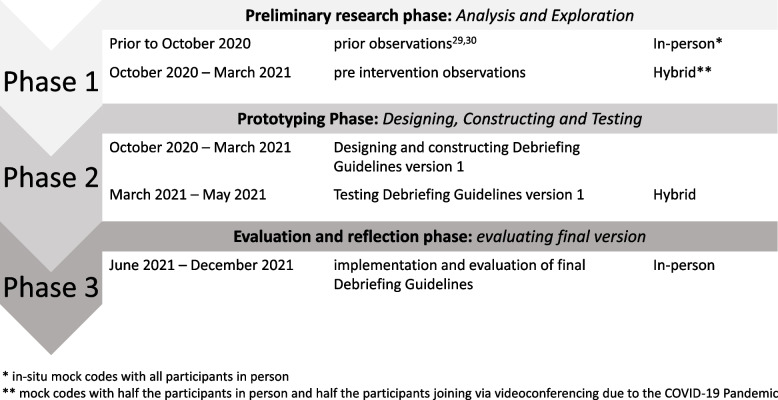
Timeline of our educational design research

Educational design research takes place in three phases: [54] (1) a preliminary research phase focused on analysis and exploration. During this phase, the problem that the design will address is more closely examined, relevant educational theories are identified, and guiding design principles are developed; (2) a prototyping phase focused on design, construction, and testing. During this phase, the intervention is developed based on educational theory and corresponding design principles; the intervention is then tested and modified based on empirical findings; and (3) an evaluation and reflection phase focused on the evaluation of the final version of the intervention. An overview of the three phases with a timeline is presented in Fig. 1.

### Setting and participants

We conducted this project in two acute care units and the pediatric intensive care unit at the University of California San Francisco Benioff Children’s Hospital, San Francisco (UCSF BCHSF), in the setting of an established ISBTT program described in a prior publication [42]. In these units, ISBTT sessions occur monthly. Participants consist of nursing staff and physicians (residents, fellows, attending physicians) as well as clinical pharmacists, pharmacy students, medical students, and respiratory therapists. All are assigned roles according to their professional positions. The sessions follow a structured format: after a short pre-briefing in which facilitators orient the participants to the mannequin and the purpose of the training, two scenarios (5–10 min) are each followed by a semi-structured group debriefing (approximately 20 min) for a total of 60 min per session. Scenarios vary across units depending on the typical patient population in that unit, but all scenarios have a decompensating patient that requires activation of the emergency response team. Learning objectives focus on the recognition and clinical management of a decompensating patient, and teamwork and communication. Each session is facilitated by two nurses and two physicians, reflecting a co-debriefing model of facilitation [52].

Prior to the current project, facilitators in the program were trained through a 4-h workshop on a participant-centered approach and principles of debriefing with good judgment [48]. During the workshop, they also reviewed the overall learning objectives of the ISBTT program, which centered on crisis resource management and essential elements of teamwork as outlined in TeamSTEPPS [3]. Facilitators were encouraged to collaborate and actively participate in pre- and debriefing, but it was left to their discretion how to divide tasks during pre- and debriefing. Facilitators’ experience ranged from < 1 year to 10 years.

Due to social-distancing requirements during the COVID-19 pandemic, we conducted a hybrid simulation during part of the prototyping phase, with some participants joining in person and others via videoconferencing (Fig. 1).

The UCSF Institutional Review Board approved this as an exempt study (IRB Number 17–23,980).

### Data collection

We collected two sets of data during the study: (1) video recordings of ISBTT sessions (including the pre- and debriefing) throughout the study period and (2) audio recordings of interviews with facilitators during the implementation phase. In addition, one investigator (NB) attended all ISBTT sessions and kept field notes that further augmented the data from video recordings. For facilitator interviews, we created a semi-structured interview guide to obtain facilitators’ feedback on the debriefing guidelines and to identify any challenges encountered with the new guidelines. Interviews were conducted by one of four researchers (NB, MJ, MN, and LT) with 1–2 facilitators at a time, lasted up to 30 min each, and were audio-recorded and professionally transcribed with the removal of identifying information. To minimize potential perceived power differentials between interviewer and facilitator, interviews were conducted by either a professional concordant member of the research team or by a PhD researcher. We transcribed the recordings of all interviews and ISBTT sessions.

### Qualitative data analysis

We used two different approaches for the analysis of our qualitative data: thematic analysis and qualitative content analysis [55–57]. We used thematic analysis [55] to examine common themes based on the review of the video-recorded pre- and debriefings, observation notes, and interview transcripts. Two members of the research team not directly involved with any of the simulations (NB and SVS) individually coded the data and generated a codebook through an iterative process. They met frequently to discuss the coding, reconcile differences, and generate themes from the data. The final themes were reviewed with the entire research team to ensure a shared understanding of the data set and its interpretation.

We used qualitative content analysis [56, 57] of video-recorded debriefings as a means to triangulate the data from our thematic analysis. To this end, we examined both the frequency of contributions made by nurse and physician facilitators and the content of their contributions. We defined a contribution as any question or statement posed by a facilitator on a discrete topic. We created a coding scheme that categorized facilitator contributions as either scripted (i.e., debriefing guidelines included a prompt for a facilitator to speak), prompted (i.e., a different facilitator asked the facilitator to speak), or spontaneous. To code pre- and debriefing content, we defined four categories: (1) logistics/expectations, (2) medical management, (3) communication and teamwork, and (4) power dynamics and hierarchy. We came to these four categories based on the initial review of two video-recorded sessions not included in the final analysis by three investigators (AW, NB, and SVS). One investigator (AW) subsequently coded all ISBTT sessions included in the comparison with these coding schemes, using web-based video annotation software (Vimeo.com Inc NY, NY). A second investigator (SVS) reviewed this coding for accuracy, and the two investigators discussed and reconciled any discrepancies in interpretation.

### Researcher reflexivity

To ensure our team had a diversity of perspectives, our research team consisted of nurses (LT, MN, AL), physicians (MJ, JE, DF, SvS), and a sociologist (NB), all had prior experience with interprofessional team training. AL, NB, MJ, and SvS also brought relevant research expertise to the work. Three members (AL, DF, and NB) of the team were not directly involved in participating, facilitating, or administrating our ISBTT, and one author was not at our institution (AL). The authors who acted as facilitators in the program were also observed as part of the study; their experiences as facilitators may have influenced their interpretation of the data. On the other hand, the authors who were not directly involved with the ISBTT program may not have had the necessary contextual information to allow for an accurate interpretation of findings. We paid attention to this in our assignment of the research team members to data analysis tasks, ensuring balanced representation as well as multiple levels of review of results to enhance the trustworthiness of our findings.

## Results

In this section, we will discuss the results of the three phases of EDBR: preliminary, prototyping, and evaluation phase.

### Preliminary research phase: analysis and exploration

For this phase, we reviewed the literature to examine how published ISBTT programs approach learning within the interprofessional context and to identify relevant theories that could inform the current project. Our review of published ISBTT programs (under review), guided our selection of two theoretical frameworks relevant to the interprofessional learning context: the social identity framework [58, 59] and transformative learning theory [60].

The social identity approach, which includes theories on social identity and self-categorization, is helpful to understand how interprofessional dynamics impact the ways in which individuals from different professions perceive and respond to each other [58]. This theoretical framework conceptualizes human behavior as having both interpersonal and intergroup aspects, with social identity defined as a person’s self-concept derived from perceived membership to a particular group. This self-categorization leads to perceptions of an “in-group” versus an “out-group,” with the in-group generally receiving more favorable consideration than the out-group [59]. Applied to teamwork in health care, the social identity approach helps explain tensions between professions based on factors such as stereotyping, hierarchy, and contextual as well as temporal changes in self-categorization.

Transformative learning theory defines learning as the social process of constructing and internalizing a new or revised interpretation of the meaning of one’s experience as a guide to action [61]. Transformative learning requires that learners hear and question alternative viewpoints; critically analyze their own assumptions, beliefs, and values; and consequently, shift their own perspectives. Perspective taking in particular has the potential to lead to impactful learning in the interprofessional context as it can help overcome bias and misunderstanding that often arise as a result of siloed training and stereotyping [60].

In addition to the discussion of educational theories that can inform ISBTT, the literature over the last decade has seen a rich discourse on how interprofessional education (IPE) and collaboration can be optimized. From this literature, we selected two perspective papers containing recommendations particularly salient to ISBTT. Paradis and Whitehead in a reflection on the history of IPE and its shortcomings encourage IPE educators and programs to engage with theory and to explicitly address power and conflict in the healthcare system and healthcare encounters [13]. Bainbridge and Regehr commenting on the difference between idealized visions of interprofessional collaboration and actual practice pointed out that any educational effort that aims to change long-established patterns of interactions between social groups needs to target both individuals and their environment [62]. To address this challenge, Bainbridge and Regehr recommend building social capital and perspective-taking, which allows team members to develop relationships with each other by creating goodwill and a safe space to interact (social capital) and an in-depth understanding of how others perceive the problem at hand and, as a result, the solutions offered (perspective taking). In addition, they suggest that negotiation of priorities and conflict management should be integrated into interprofessional education to mitigate existing differences effectively [62].

Informed by this literature review and our prior work, we developed five design principles for interprofessional debriefing guidelines (Table 1) through iterative discussions among the research team.

**Table 1:** Initial design principles

Design principle	Rationale/theoretical basis
• Interprofessional collaborative approach to facilitation	Model desired behaviors; increase psychological safety for participants, social identity theory [47, 48]
• Expect active participation by all	Transformational learning theory [49]
• Focus on teamwork and collaboration	Principles of interprofessional education, recommendations by Paradis [50]
• Encourage perspective taking	Transformational learning theory, recommendations by Bainbridge and Regehr [51]
• Make issues of hierarchy and power explicit	Recommendations by Paradis [50]

### Prototyping phase: designing, constructing, and testing

Our initial set of design principles informed the first version of structured pre-briefing and debriefing guidelines for facilitators. Table 2 delineates the elements of the guidelines mapped to the design principles, as well as changes made iteratively throughout the prototyping phase. The guidelines assign roles to facilitators from different professions and contain scripted text to guide pre- and debriefing. Debriefing questions and prompts explicitly address teamwork, collaboration, hierarchy, and power, and promote perspective-taking. We also provided practical and general tips for debriefing, including timekeeping, note-taking, and approaching learners with curiosity. We discussed the first version of the guidelines with the research team and made minor changes in wording based on feedback.

**Table 2:** Recommended changes to the first version of guidelines by design principle

Design principle	Guideline element(s)	Observations	Recommendations
• Interprofessional collaborative approach to facilitation	• Assigned roles and scripts for RN and MD facilitators in pre-briefing and debriefing	Works well in pre-briefing, debriefing is still mostly physician-led	• More prominent role for RN facilitators early in debriefing • Ask MD facilitators to review their own videos and reflect on creating space for RN facilitators
• Expect active participation by all	• Discuss as a ground rule in pre-briefing • Assign participants active roles and observers • Invite participants and observers to speak in debriefing	Variable participation in debriefing, if RN facilitators have a clear presence, RN participants appear to speak more	• Create more space for RN facilitators (see above) • Explicitly invite RN participants to speak
• Focus on teamwork and collaboration	• Highlight the goal in pre-briefing • Focused questions in debriefing	Most debriefs focus primarily on teamwork and collaboration. Some MD facilitators comment there is a need for discussion of medical content	• Early in the debrief ask for a mental model regarding patient’s medical condition • Develop asynchronous method for in-depth medical content knowledge sharing
• Encourage perspective taking	• Discuss as a ground rule in pre-briefing • Focused questions in debriefing	Doesn’t happen consistently	• If mental models are incongruent, explore why the team thinks others have a different model • Include example questions in guidelines to promote perspective-taking
• Make issues of hierarchy and power explicit	• Set expectations in pre-briefing • Focused questions in debriefing	Variable whether this is addressed, participants don’t always seem comfortable and facilitators vary in comfort and skill. Some feel the framing in the pre-briefing is too direct	• Change wording in pre-briefing to explicitly acknowledge the tension between experience versus position/role, without necessarily using words like hierarchy and power • Example questions with open-ended framing • Elicit examples from real life

The implementation of the guidelines included a 3-step training process to train all 26 facilitators in our ISBTT program (18 nurses, 8 physicians) on the use of the new debriefing guidelines. First, all facilitators reviewed an asynchronous video presentation explaining the rationale for our intervention and introducing the new facilitation guidelines. Second, we conducted 1-h interprofessional facilitator training that allowed for role-play of pre-briefing and debriefing. We then provided a debrief with facilitators after their first use of the guidelines to help identify areas of improvement and provide feedback to the facilitators. MJ, JE, LT, and MN conducted all facilitator training.

Once all facilitators completed the training, we piloted the debriefing guidelines in nine ISBTT simulations over a 3-month period between March 5, 2021, and May 25, 2021 (Fig. 1). Two researchers (NB and SvS) analyzed data from video-recorded sessions and interviews with facilitators conducted during this period to gain insights into the use of the guidelines.

Based on their analysis, these researchers proposed recommendations for adjustments to the guidelines, summarized in Table 2. After discussion with the entire research team, we created a new version of the guidelines which was implemented from June 4, 2021, onwards. We conducted a similar process to examine the use of guidelines during the next 3 months (Fig. 1) through a review of 7 video-recorded simulation sessions and 8 interviews with 14 facilitators. The same two investigators (NB and SVS) analyzed the data, and while they observed variable implementation of the guidelines, they did not feel additional changes to the guidelines were warranted. Rather, they recommended ongoing facilitator training and feedback to optimize their comfort and facility with the guidelines, which we worked on implementing after the completion of this study. The final version of the guidelines is presented in Additional file 1.

### Evaluation phase: comparing the conversation before and after the intervention

#### Thematic analysis

To explore the impact of the guidelines, we conducted a thematic analysis of all qualitative data collected through a review of video-recorded sessions and facilitator interviews before and after the implementation of the guidelines. Through this analysis, we identified two major themes: (1) changed conversation during the debriefing and (2) improved interprofessional learning. For each of the major themes, we describe the differences in our observations before and after the implementation of the guidelines. We summarize these findings below and provide illustrative quotes in Table 3.

**Table 3:** Themes with representative quotes

Theme	Representative quotes
Changed conversation during the debriefing	
*Before the implementation of guidelines*	The physician facilitator talks about how one can be hesitant to administer intramuscular epinephrine and discusses the distinction between an anaphylactic reaction and an allergic reaction. Then they ask the resident who was team leader in the scenario what her reasoning process was and she answers. The physician facilitator starts a discussion about oxygen delivery, nurses explain what they have access to and describe the tools they have available. (Observation Pre-implementation Session 5)
	A participant brings up noticing a discrepancy between the medication dose initially ordered and eventually given (due to two different physicians giving orders, though the participant does not mention it). The participant says: “Maybe I should have said something.” The nurse facilitator says that speaking up is definitely useful, but there is no discussion of why the participant did not speak up. (Observation Pre-implementation Session 2)
*After the implementation of guidelines*	“I think sometimes it gets into like very teachy, especially with, depending on the residents that are on, they ask a lot of clinical questions with the physicians. So sometimes this [the guidelines] helps kind of say, “Okay, we can talk about this, but not right now. We're going to focus on these points right now with the whole team" (Interview 1, Nurse Facilitator)
	The physician facilitator gives feedback about the interactions between the bedside nurse and resident in the scenario. She says the resident was reasoning out loud, the bedside nurse offered suggestions, so she asks why they did so. The bedside nurse says he was just looking at what was happening, recommended things he was sure of. The physician facilitator asks the bedside nurse if there is anything that made him feel safe to make suggestions. He hesitates, then says “it was just the way that we were talking to each other, it didn’t feel like, as if he’s coming into the picture… And just thinking about past actual codes, where you feel like it’s an inconvenience that the code is happening or that you called the doctor. Just in this dynamic, in this specific scenario, I felt comfortable to say ok, this is what is going on.” (Observation Post-implementation Session 5)
	Nurse facilitator talks about power and hierarchy, uses her own words and gives examples of how to create collaborative environments and speak up. (Observation Post-implementation Session 1)
	[…] “bringing attention to the power and hierarchy actually has been really useful in my opinion. People have had multiple opportunities to bring that up in a really organic way because it comes up every single time. Like someone being hesitant to voice their opinion. And we're like, all right, let's unpack this, let's talk about why. Even though the script feels kind of stiff when you're doing it at first, the content that comes up naturally out of mock code just leads you to go into that a lot more naturally.” (Interview 9, Nurse Facilitator)
	Nurse observer: “Can I just thank you, and everybody actually, for acknowledging the power dynamics. Because again, in this group, this is a very strong group of nurses, and I know, I’ve been working with these guys [points to the medical team] for three days and I know the resident from before, so I think it’s not necessarily at play in this room, but it will be in real life. And so I think it’s really important to acknowledge that and to talk about it because that will help to bring that down outside and in the real world.” (Observation Post-implementation Session 4)
	“And [the guidelines] gives good probing questions, I saw RN Facilitator 7 used it to ask about teamwork and power and dynamics, which I don't know if we would feel as comfortable talking about without having those words on a paper to read.” (Interview 7, MD Facilitator)
Improved interprofessional learning	
*Before the implementation of guidelines*	The physician facilitator starts a discussion about oxygen delivery. Nurse participants explain what they can typically do, go through the emergency equipment they have access to give oxygen to patients. The nurse facilitator adds more specifics about what nurses are allowed to do and what they should know. (Observation Pre-implementation Session 5)
*After the implementation of guidelines*	The physician facilitator says that in this scenario, what was going on with the patient was less clear than in the first scenario, so how do people feel about teamwork and hierarchy? The senior resident answers that in situations like that she always sees the bedside and charge nurses as her main resources, so she turns to them for questions about the patient and if she is not sure what to do. She says: “Often, the nurses will say ‘are you sure?’ and I’m like, ‘no!’” (…) The nurse facilitator says that she also noticed that the senior resident asked the room for ideas and suggestions, talks about how people often expect the physician to know everything but asking the room was good to get input and elicit speaking up from team members. (Observation Post-implementation Session 5)
	The resident talks about when they were trying to decide whether to run or push the medication, says that she was about to make a decision when the nurses asked if she was sure, which she says was good, but she says she also realized that the nurses would have done whatever she decided despite knowing that she was wrong. The physician facilitator asks questions to elicit more perspective from the resident, gives her an opportunity to explain more. The resident answers: “I fully trust my nurses, like, yeah, let’s do what you have been doing for the last 20 years, I’ve only been here for two.” (Observation Post-implementation Session 7)
	Nurse facilitator asks if people noticed any communication breakdowns. Someone answers they struggled with medications, explains what he did to communicate better since information sometimes didn’t make it clearly to the code cart. Pharmacist says she could not find labels so when they were asking for medication she was holding what they wanted but could not give it because she did not have labels. (Observation Post-implementation Session 2)

- Changed conversation during the debriefing:
  - *Teamwork and communication*
    Prior to implementation of the guidelines, physician facilitators often mentioned teamwork and communication during the pre-briefing, explaining that the purpose of ISBTT is to practice teamwork and that they would address these topics during the debriefing. Indeed, in most debriefings, physician facilitators discussed examples of good teamwork or communication that occurred during the scenario. On occasion, facilitators prompted conversations of suboptimal communication or ineffective teamwork, but they tended to address these shortcomings at a superficial level without delving into why they occurred.
    After implementation nurse facilitators were more involved in discussions of teamwork and communication, and both facilitators and participants seemed increasingly more comfortable with probing questions about challenges. While this sometimes led to open discussions about opportunities for improvement, such probing questions were frequently followed by compliments or explanations for the challenges experienced which limited further discussion.
  - *Power and hierarchy*
    Prior to the implementation of the guidelines, a few physician facilitators occasionally acknowledged noticing power and hierarchy and their impact on teamwork and communication. When this occurred, the discussion was brief and limited to generic statements about the importance of speaking up for patient safety, especially questioning decisions by physician team leaders.
    Power and hierarchy were more frequently discussed after implementation of the structured guidelines, with nurse facilitators bringing these topics up in almost every session as prompted by the guidelines. Initially, we observed variability in facilitators’ adherence to this part of the script, sometimes noticing hesitancy and sometimes they simply skipped over the prompts. In interviews some facilitators acknowledged feeling challenged by these discussions, with a few expressing worries about creating a negative atmosphere if power and hierarchy were brought up too often or too early. As time went on, facilitators more consistently followed this part of the guidelines and started to paraphrase in their own language rather than using the scripted text. They more frequently included clear examples from scenarios and references to real life experiences. Participants initially did not always answer the questions about hierarchy and power or came up with answers that said they had not noticed any issues. Over the course of the project period, participants more frequently engaged in discussions about power and hierarchy, recognizing that these factors impacted the simulated scenario as well as situation in real life.  Some participants expressed appreciation for the explicit addressing of power and hierarchy during the debriefing. However, there was notable variability between different facilitators and sessions, and we noted missed opportunities to delve into how power dynamics may prevent clinicians from asking for help or admitting uncertainty.
  - *Clinical management*
    Prior to implementation of the guidelines, discussion on clinical and technical skills were led by physicians. This typically took the form of knowledge transfer between facilitators and team members. After implementation of the structured guidelines, clinical aspects of scenarios were still a major component of debriefing discussions, but nurses were more involved in the discussion. Nurse facilitators more frequently brought up questions about medical management, and physician facilitators increasingly invited nurse facilitators and members from other professions (i.e. pharmacy) to provide input. Discussions about medical management were also less physician focused and more frequently occurred in the context of discussing teamwork. Nurse facilitators viewed this change positively and reported that it helped all participants benefit from the ISBTT sessions. Some physician facilitators, however, expressed concern about residents’ learning as they feared the new guidelines did not leave enough room for discussion of clinical aspects of scenarios.
- Improved interprofessional learning
  Prior to implementation of the structured guidelines, interprofessional learning mostly occurred through participants’ learning about each other, for example, nurses explaining their role in emergencies. These discussions were usually prompted by questions from participants or facilitators. On occasion, physician facilitators described nurses’ roles to the group, without letting nurses speak for themselves. We also observed participants learning from each other, when they explained their rationale for certain actions during the scenario to the group. Typically, this occurred when residents explained their clinical decision making to the rest of the team.  We rarely observed evidence of perspective taking.
  In contrast, after implementation of the structured guidelines interprofessional learning increasingly included perspective taking, through discussions focused on both the nursing and physician perspective. Rather than simply justifying their actions, residents described feeling vulnerable and lost in some scenarios, acknowledging their reliance on the more experienced nurses on the team. This prompted broader conversations about how participants may collaborate to help each other as well as discussions about the different sources of power that create imbalances between team members. For example, participants often contrasted residents’ leadership position and lack of experience with nurses’ lack of a leadership position and breadth of clinical experience.
  As a whole, facilitators and participants shared increased recognition of the value of learning from and about others, including understanding each person's role, their limitations, as well as barriers to communication due to power dynamics.

## Qualitative content analysis

To enhance the trustworthiness of our thematic analysis, we examined whether the guidelines impacted conversations in terms of participation in and content of debriefing before and after implementation of the guidelines through qualitative content analysis of video-recorded simulation sessions. To get a sense of the magnitude of the noted changes, we quantified contributions in each of the categories prior to the intervention and after the implementation of the final guidelines (Table 4).

**Table 4:** Qualitative content analysis of facilitator contributions to pre- and debriefing before and after implementation of guidelines

	Nurse facilitators		Physician facilitators		Total	
	Before	After	Before	After	Before	After
Type of contributions						
All	24 (17%)	100 (41%)	119 (83%)	141 (59%)	143	241
Scripted	0 (0%)	64 (45%)	0 (0%)	40 (17%)	0 (0%)	104 (43%)
Prompted	0 (0%)	4 (2%)	6 (4%)	11 (5%)	6 (4%)	17 (7%)
Spontaneous	24 (17%)	32 (13%)	113 (79%)	90 (37%)	137 (96%)	122 (51%)
Content of contributions						
Logistics/expectations	7 (5%)	32 (13%)	24 (17%)	40 (17%)	31 (22%)	72 (30%)
Medical management	8 (6%)	37 (15%)	59 (41%)	49 (20%)	67 (47%)	57 (24%)
Communication and teamwork	9 (6%)	24 (10%)	34 (24%)	49 (20%)	43 (30%)	73 (32%)
Power dynamics and hierarchy	0 (0%)	7 (5%)	2 (1%)	12 (5%)	2 (1%)	19 (8%)

Data points represent the number of contributions by facilitators in each category across all sessions in a time period (before vs after implementation of the structured guidelines, 7 sessions in each period) and percentages are calculated with all contributions in time period as denominator

Prior to the implementation of guidelines, physician facilitators did the majority of the talking, providing over 80% of all contributions during pre- and debriefing. With the implementation of the guidelines, nurse facilitators’ contributions increased. While scripted contributions (as dictated by the guidelines) accounted for a large proportion of this increase, we also observed more prompted questions (i.e., facilitator is asked to speak by the other facilitator or a team member) and spontaneous contributions from nurse facilitators. We also noted a shift in the content of contributions made by both nurse and physician facilitators (Table 3).

## Discussion

In this work, we described how we designed and implemented guidelines for pre- and debriefing for ISBTT. We were able to successfully integrate these guidelines into an existing ISBTT program and adapt it to the needs of the facilitators and our context. Our educational design research approach allowed us to ground the guidelines in theory and ensured we took an iterative approach taking input from stakeholders into account throughout the design process.

Interprofessional education is often defined as learning “with, from and about each other” [63]. After implementation of our new facilitator guidelines, including training and discussion with facilitators, we noted increased interprofessional learning, exemplified by participants’ learning more *from* members of other professions, as well as increased learning *about* each other’s roles, responsibilities, and perspectives. This change was seen in both directions between physicians and nurses, which may have led to more in-depth discussion about how and why effective collaboration and teamwork can be difficult. Previous studies have investigated teaching and learning specific skills aiming to address power discrepancies in healthcare teams, such as speaking up for patient safety [23–25]. However, many of these prior studies were conducted with one profession as learners, not addressing the complexities of interprofessional education.

Prior to the implementation of these guidelines, our program utilized interprofessional co-debriefing, defined as “more than one facilitator conducting a debriefing session, in which each facilitator comes from a different healthcare professional background, for example, nursing, medical, physiotherapy, or others” [64]. Despite this, the physician facilitators in our program were the dominant voices during simulation sessions, similar to what has been observed in other ISBTT programs [16]. These dynamics mimic what is often seen in the clinical setting [62, 63]. Existing literature on co-debriefing suggest the use of two structured approaches to co-debriefing in simulation-based education: “follow the leader” and “divide and conquer” [52]. Our scripted guidelines utilized the “divide and conquer” technique to assign specific roles to facilitators based on their professional background, rather than the “follow the leader” model which was used prior. The use of this technique in the interprofessional setting appeared to empower nurse facilitators to take the lead and share their expertise during the pre- and debrief. At the same time, it seemed to limit opportunities for physicians to dominate, thereby countering traditional hierarchical interprofessional dynamics. This redistribution of facilitator roles can lead to changes in power dynamics since it allows for nurses to demonstrate more expertise, which comes with its own form of power [65]. In addition, this redistribution of roles can provide modeling for participants and, as a result, change their attitudes and behaviors [66]. Viewed through the lens of social identity theory, this modeling of shared facilitation can set the social norm for the simulation session and potentially shift the mental model among team members from in-group/out-group thinking along professional boundaries to one where all professions are part of one in-group: the interprofessional team [59]. Continued research is needed to better understand how power and hierarchy among interprofessional co-debriefers influences interprofessional learning among the interprofessional team [64].

Our data suggests that perspective-taking did not come naturally to our facilitators and participants in ISBTT debriefing, yet it is clear from the literature that perspective-taking can promote cooperation, coordination, helping, and conflict management [67–69]. This lack of comfort with perspective-taking in the simulation setting is not surprising, as many situational factors can affect an individual’s ability to engage in perspective-taking, including diminished cognitive resources during times of stress and anxiety, such as facilitating a simulation debrief [56]. Looking at the work of a facilitator through the lens of cognitive load theory, [70] the use of scripted facilitator guidelines could potentially help relieve extraneous load and increase germane load by assigning roles and providing language to ask challenging questions around power, hierarchy, and perspective-taking.

The implementation of debriefing guidelines coincided with an observed increase in the frequency of discussions around teamwork, hierarchy, and power. Yet, our data shows that facilitators varied in their ability and comfort in leading such conversations, highlighting the need for ongoing facilitator training. While our one-time training incorporated many aspects of best practice in faculty development for simulation [71], including teaching multiple debriefing methods, deliberate practice, and feedback, our experience indicates that facilitators would benefit from continued opportunities to receive feedback. As a future area for development, we will be developing a peer-coaching facilitator program to provide individualized support for our facilitators [72].

Of note, some physician facilitators in our program felt that a strong focus on teamwork and collaboration limited the space dedicated to discussion of medical learning points. To address this, we are developing asynchronous debriefing materials that transmit such information to participants. At different institutions, learner needs may differ. Thus, a thorough exploration of needs and expectations and ensuring that uni-professional learning objectives do not get lost is important in adapting our guidelines elsewhere. This is just one of many factors to consider before implementing our approach to debriefing at other institutions. It should be noted that in our context, time for ISBTT is limited, and increasingly so, because of the many competing demands on our healthcare professionals and limited funding to ensure they have sufficient time to participate and/or facilitate ISBTT. We therefore designed our guidelines for sessions that take place over only 1 h, recognizing that longer pre- and debriefs may be desirable, and even necessary, in different contexts. We also want to acknowledge that a different approach to ISBTT by itself will likely never be sufficient to lead to major changes in interprofessional dynamics, yet we are optimistic that it can be one of the interventions that contribute to the changes necessary to optimize interprofessional collaboration.

This work has important limitations that should be considered before implementing our guidelines at other institutions. First, because EDR occurs in natural settings, the outputs of EDR are context-dependent and influenced by many variables exist that cannot be controlled for [54]. Each institution will have different variables, and this should be taken into consideration when developing and implementing scripted facilitator guidelines. For instance, our data demonstrates that there was variable comfort in discussing power and hierarchy in the interprofessional simulation setting. This may not be the case in certain environments based on local facilitator training and experience. Second, our study took place during the height of the pandemic, leading to a mix of hybrid and in-person mock code simulation sessions, which may have influenced the outcomes. Additionally, due to the pandemic, increased burnout, and many challenges in the clinical setting, information on the impact on participants’ perceptions, attitudes, and behaviors is missing from this work. Third, we conducted this project at a single institution, a children’s hospital, and whether similar uptake will occur in different settings and contexts is not clear. Third, we did not modify the scenarios used in our simulation to specifically bring out interprofessional dynamics such as power and hierarchy. Doing so may have brought these topics more into focus and may have facilitated meaningful discussions during the debriefing. Lastly, Educational Design Research, while attractive for designing and piloting interventions in complex real-life environments, is not necessarily suited to study whether an intervention has a statistically significant impact on measurable outcomes. The data we present as part of the qualitative content analysis should be reviewed with this caveat in mind, as it is meant as illustrative rather than quantifiable evidence of the intervention’s impact, which will require interventional studies with appropriate sample size. Due to the fact that our EDR-based study took place in a real-world environment, rather than in the controlled setting of a simulation laboratory, we recognize other factors outside of the implementation of scripted guidelines could have impacted our findings. Future work could involve a multi-institutional to examine what factors contribute to changes in conversation during interprofessional debriefs in different contexts and settings, to further help others optimize ISBTT.

## Conclusion

We successfully implemented new guidelines for pre-and debriefing of ISBTT, which aim to create an interprofessional approach to facilitation and promote discussions about teamwork, collaboration, hierarchy, and power as well as perspective taking. While we recognize that our guidelines may need adaptation for different contexts, we hope our work will encourage others to consider the various theoretical underpinnings that determine successful interprofessional collaboration when designing or improving their ISBTT programs. We believe that relying on simplistic approaches that overlook the complex social dynamics of interprofessional teams will not be sufficient to change the status quo and thus limit our ability to truly optimize interprofessional collaboration.

## Supplementary Information

Additional file 1. Final version of the ISBTT guidelines.

## Data Availability

The data used and/or analyzed during the current study are available from the corresponding author on reasonable request.

## Abbreviations

ISBTT: Interprofessional simulation-based team training
EDR: Educational design research
IPE: Interprofessional education
