# Supplementary material for: Changing the conversation: impact of guidelines designed to optimize interprofessional facilitation of simulation-based team training
Source: Adv Simul (Lond). 2024 Oct 12;9:43. doi: 10.1186/s41077-024-00313-3 (PMC11476600; doi:10.1186/s41077-024-00313-3)
Supplement: Supplementary file 1 — Additional file 1. Final version of the ISBTT guidelines. [file 41077_2024_313_MOESM1_ESM.docx]

**Pre-Brief (5 minutes)**

Nurse Facilitator: Welcome and introductions (name and role)

Physician Facilitator: Goal

“We are doing mock codes to practice scenarios of caring for acutely ill children as a team, so we are well prepared for real-life scenarios. We will pay special attention to the dynamics within our team that help or hamper teamwork.”

Nurse Facilitator: Group Agreements

“We should set some group agreements, and I invite everyone to provide suggestions. I’ll start with one _______” [these are examples to fill in, participant provide]

- *This is a safe space:* what we do and say here stays here
- *We are here to learn:* it is okay to make mistakes
- *Everyone Participates:* If you are not actively participating you are an active observer; we learn the most if we are all engaged
- *Be Respectful:* it is okay to disagree and sometimes important to disagree, but with respectful tone, use “i-statement”
- *Assume good intentions:* everyone here is doing their best
- *Suspension of Disbelief:* the more you act how you would in real life, the more you will get out of the scenario

Physician Facilitator: Agenda for the day

“There will be two scenarios today, 5-10 mins in length with a 10-20 minute debrief after each scenario”

Nurse Facilitator: Review Mannequin

“We would like orient you to the mannequin”

- Heart and lung sounds
- Pulses (Sim Jr. left radial, brachial, carotid; SimBaby left radial, brachial, femoral)
- Fontanelle in SimBaby
- Do not put stickers on or put IVs in

Physician Facilitator: Instructions for observers

“Observers will be filling out a tool and we will ask you to provide feedback to the participants on observed behaviors and interactions”

Nurse Facilitator: Addressing power

“We also would like to recognize that hierarchy and power dynamics play a role in these mock codes as well as in real life situations. We want to remind you that we are here to work together, and that each individual brings a unique perspective. We should strive to collaborate and create an environment where everyone feels comfortable to speak up.”

**Debrief (15-20 minutes)**

Physician Facilitator: Emotional Release

- Thank and congratulate people for participating, acknowledging that its challenging; specifically, how every person experiences the session differently due to their position (ie. md/rn) and years of experience
- Reminds participants of ground rules established in pre-briefing
- Invites ALL to self-reflect

Nurse Facilitator: Assess mental model

- Directed to Charge RN: What was going on with this patient? Directed to MD leader: What did you think was going on?
- Directed to All: Did others think similarly?
- Comment if models match or differ
- Differences in the mental models: question why this might be, explore why the charge nurse/md might have different viewpoints (SEE POTENTIAL QUESTIONS below)

Physician Facilitator: Address Clinical Learning Objectives

- With input from all facilitators, clarifies any main teaching points from general and specific mock code objectives (e.g. medical management, workflow around codes, etc).

POTENTIAL QUESTIONS (to be asked by nurse/MD facilitator) ~ Bolded ones are highly recommended:

- Did factors related to hierarchy or power dynamics play any role?
- Does being a nurse or resident influence how you respond in the scenario?
- What do you think you would have done in X’s role, can you imagine how this was for that person?
- How did you feel about the teamwork in this scenario? How did this impact the mental models?
- Did you notice any break in communication/ collaboration? If so, why do you think they occurred?
- Have you been in a similar situation that you didn’t feel you could speak up and why was that (in real life)
- Open ended Qs: Why do you think you didn’t say anything?

Nurse Facilitator: Closes the debrief examples of ways to close

- Summarize how observed behaviors impacted scenario flow and outcome
- Ask participants to share one thing they will take away from this session back to the bedside regarding teamwork


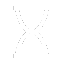

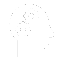

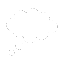

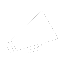

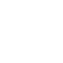

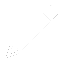


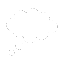

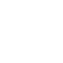

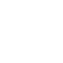

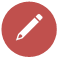

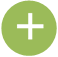

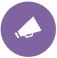

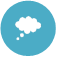

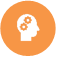

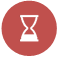


**KEEP TRACK OF TIME:**

GENTLY INTERRUPT PEOPLE WHO TAKE UP MORE SPACE

**MORE PERSPECTIVE TAKING PROMPTS:**

*WHAT WOULD YOU HAVE DONE IF YOU WERE IN THE SHOES OF X...*

**BE CURIOUS:**

BEING CURIOUS ABOUT PEOPLE’S THOUGHTS, MOTIVATIONS

**TAKE NOTES:**

EVEN IF JUST A QUICK SCRIBBLE TO REMIND YOURSELF OF THINGS THAT YOU MAY WANT TO ADDRESS DURING THE DEBRIEF

**INVITE:**

INVITE THOSE WHO ARE NOT SPEAKING TO SPEAK

**COUNT TO 10:**

SOMETIMES SILENCE IS AWKWARD AND WE JUMP TO FILL THE VOID
